# [Corrigendum] Epithelial protein lost in neoplasm-α (EPLIN-α) is a potential prognostic marker for the progression of epithelial ovarian cancer

**DOI:** 10.3892/ijo.2026.5874

**Published:** 2026-03-18

**Authors:** Rong Liu, Tracey A. Martin, Nicola J. Jordan, Fiona Ruge, Lin Ye, Wen G. Jiang

Int J Oncol 48: 2488-2496, 2016; DOI: 10.3892/ijo.2016.3462

Following the publication of the above article, an interested reader drew to the authors' attention that, regarding the cell invasion assay experiments shown in Fig. 5A on p. 2493, the 'WT' and 'pEF6' data panels were strikingly similar, albeit the coloration of the images differed slightly from each other, suggesting that these images were derived from the same original source. A second reader also noted that, concerning the scratch-wound assay experiments shown in Fig. 6B, the images for the WT and pEF6 experiments at the 0 h (top panels) and 36 h (lower panels) time points contained overlapping sections (specifically, the 'WT/0 h' panel with the pEF6/0 h panel, and the 'WT/36 h' panel with the pEF6/36 h panel), suggesting that the two pairs of panels were likewise derived from the same original sources.

Upon examining their original data, the authors realized that these figures had been inadvertently assembled incorrectly. Revised versions of Figs. 5 and 6, now showing the correct data for the 'WT' panel in Fig. 5A and the pEF/0 h and pEF/36 h panels in Fig. 6B, are shown on the next two pages. Note that the errors made in assembling these figures did not have a gross effect on the conclusions reported in this study. The authors thank the Editor of *International Journal of Oncology* for granting them the opportunity to publish this corrigendum. All the authors agree with the publication of this corrigendum; furthermore, they apologize to the readership of the journal for any inconvenience caused.

## Figures and Tables

**Figure 5:**
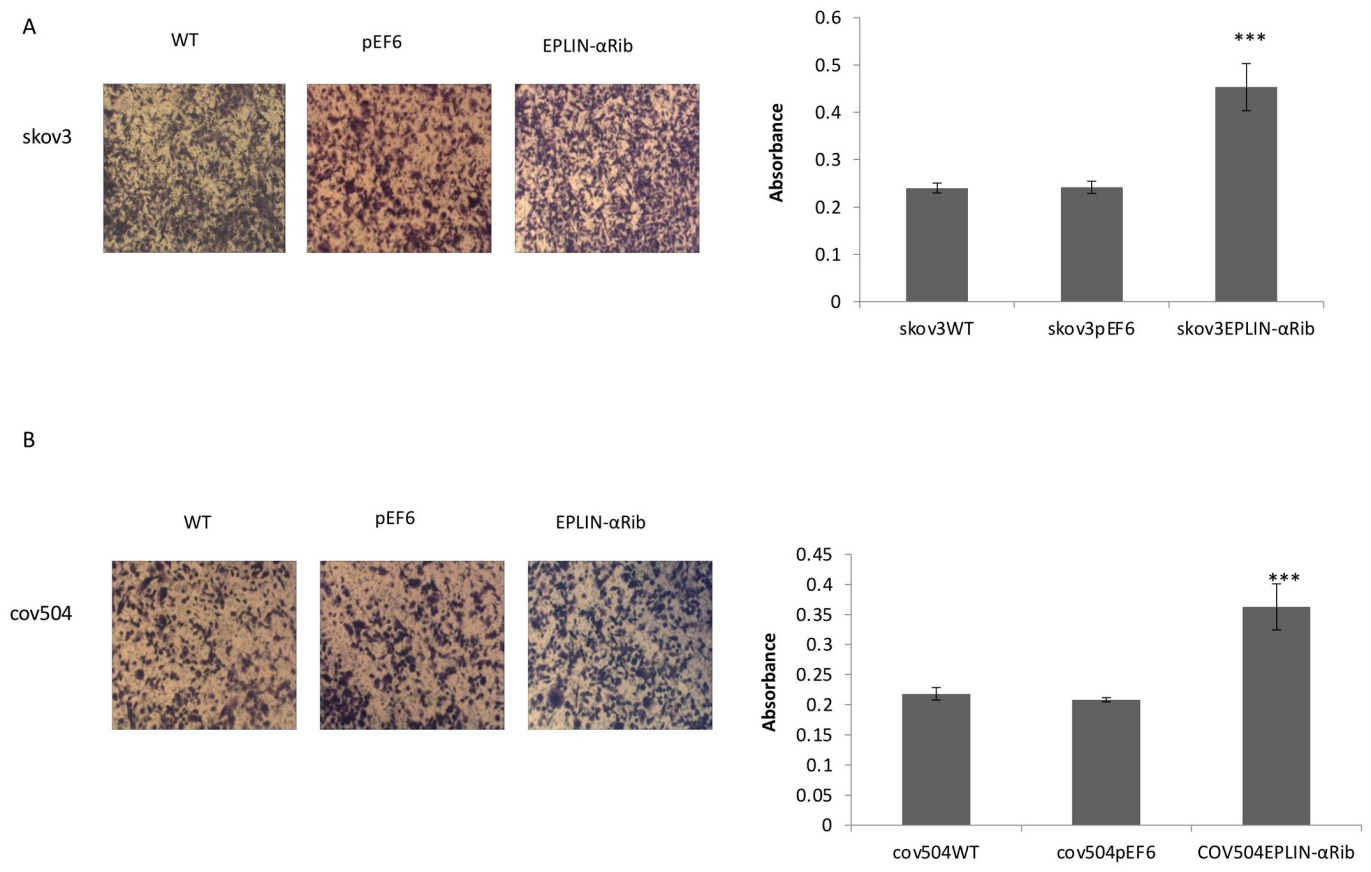
Effect of Eplin-α decreased-expression on the invasion of EOC cells. Cell invasion through Matrigel coated barrier was quantitated after fixing and staining invaded cells. Invasion capability was increased in SKOV3Eplin-α (A) and COV504Eplin-αRib (B) cells. Four fields of view/insert were photographed and counted. Images shown are from a typical field of view (×4 magnification). Data shown are the mean ± SD cell number/membrane from 3 independent experiments. t-test compared Eplin-αRib versus the controls for each cell line. ^***^p<0.001.

**Figure 6:**
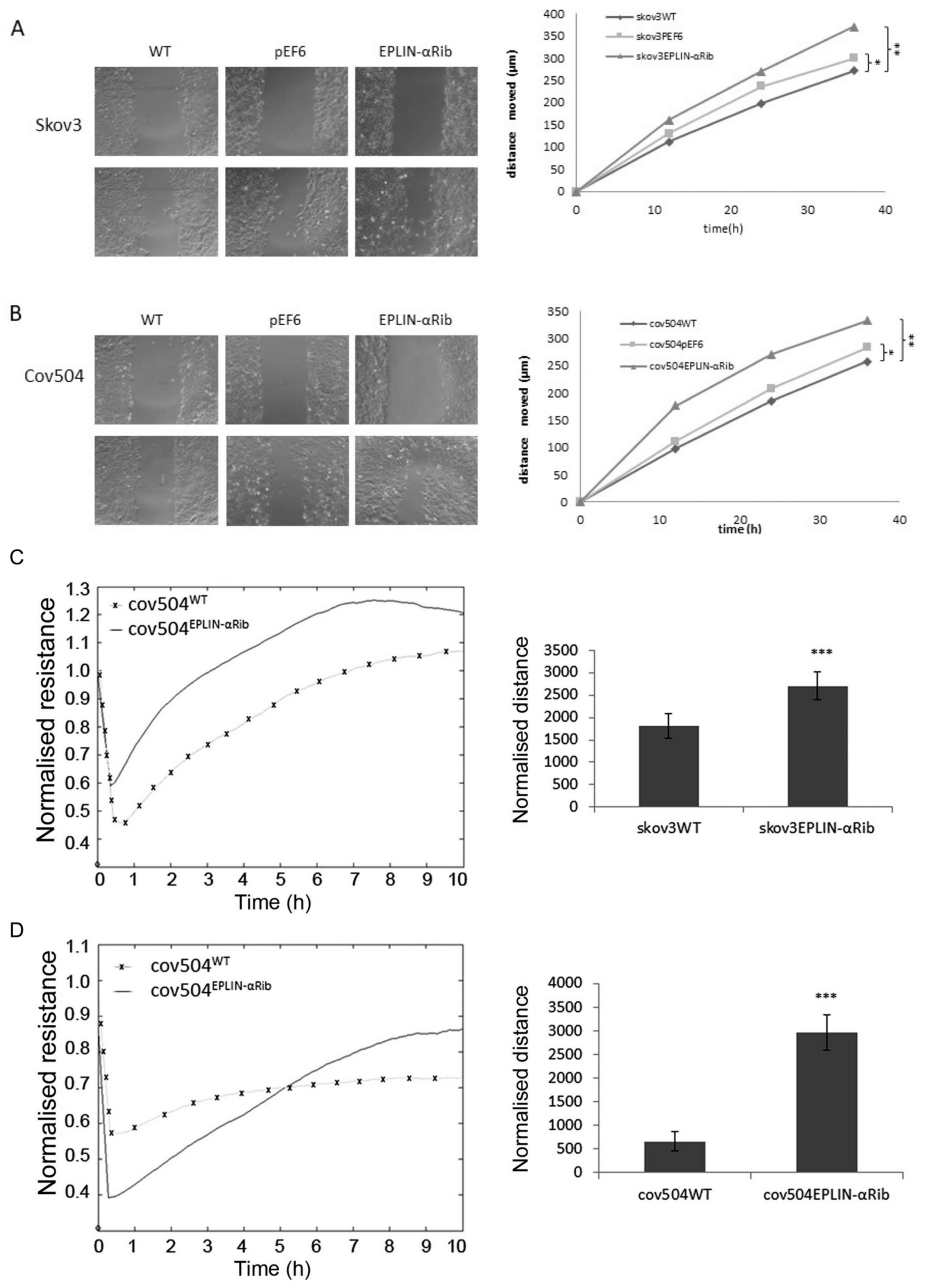
Effect of Eplin-α expression on migration of EOC cells. Confluent monolayers of EOC cells were scratched and the distance moved by cells to cover the wound was measured after 12, 24 and 36 h and compared to time 0. After 36 h, SKOV3 (A) and COV504 (B) cells with knockdown of Eplin-α protein expression all showed significantly increased migration compared to WT or pEF6 controls (t-test ^*^p<0.05 or ^**^p<0.01). Images shown are taken at 0 and 36 h are from representative experiment. The data shown are from 3 independent experiments. ECIS confirmed the increased migratory capability of SKOV3Eplin-αRib (C) and COV504Eplin-αRib (D) cells compared to the appropriate empty vector control cells. At time 0 the confluent monolayer of cells was electrically wounded and the impedance changes were recorded over 10 h as an indication of how rapidly the cells migrated to cover the wound. Data show relative change in resistance from 0 to 10 h for each cell line and are the mean of 3 independent experiments ± SD. ^***^p<0.001 comparing Eplin-αRib versus control cells.

